# Aldose Reductase: A Promising Therapeutic Target for High-Altitude Pulmonary Edema

**DOI:** 10.3390/ijms26010341

**Published:** 2025-01-02

**Authors:** Dan Song, Mengjie Wang, Xinjie Zhao, Yanru Zhang, Yiyi Zhang, Xiaohua Hao, Jialu Yuan, Haojie Tang

**Affiliations:** Key Laboratory for Molecular Genetic Mechanisms and Intervention Research on High Altitude Disease of Tibet Autonomous Region, School of Medicine, Xizang Minzu University, Xianyang 712082, China; 18780174757@139.com (M.W.); 19723032309@139.com (X.Z.); 18646783495@139.com (Y.Z.); 19723036159@163.com (Y.Z.); 18335429745@139.com (X.H.); 15047356350@139.com (J.Y.); thj1227286096@163.com (H.T.)

**Keywords:** aldose reductase, high-altitude pulmonary edema, vascular pressure, inflammatory factors, oxidative stress

## Abstract

The Qinghai–Tibet Plateau, famously known as the “Roof of the World”, has witnessed a surge in individuals traveling or working there. However, a considerable percentage of these individuals may suffer from acute mountain sickness (AMS), with high-altitude pulmonary edema (HAPE) being a severe and potentially life-threatening manifestation. HAPE disrupts the balance of intrapulmonary tissue fluid, resulting in severe lung function impairment. Current therapeutic interventions for HAPE have limitations and are accompanied by significant side effects. Aldose reductase (AR), a crucial enzyme in the polyol metabolic pathway, has been implicated in various diseases. In this study, we sought to explore the role of AR in HAPE. Utilizing both in vivo and in vitro models, we investigated the impact of AR on hypoxia-induced pulmonary edema, vascular pressure, inflammatory factors, and oxidative stress. Our findings revealed that AR knockdown mitigated hypoxia-induced pulmonary edema, decreased the expression of vascular pressure and inflammatory factors, and enhanced the expression related to oxidative stress. These results indicate that AR may serve as a potential therapeutic target for HAPE, offering a plausible pathological basis and novel drug targets for the prevention and treatment of this condition.

## 1. Introduction

The Qinghai–Tibet Plateau, with an average altitude exceeding 4000 m, is renowned as the “Roof of the World.” As the economic and technological development of Tibet Autonomous Region progresses, an increasing number of individuals are traveling to or working on the Qinghai–Tibet Plateau. Among them, a proportion of individuals, upon their initial arrival at high-altitude areas (above 2500 m), may develop acute mountain sickness (AMS). Among the severe forms of AMS, high-altitude pulmonary edema (HAPE) stands out due to its high mortality rate, rapid onset, and severe clinical manifestations [1]. Although significant improvements in medical conditions in China’s highland areas have led to a decline in the incidence and mortality rates of HAPE in recent years, untimely treatment can still result in respiratory or lung infections, shock, or even death, posing a severe threat to patients’ lives and, to some extent, hindering economic and social development in these regions [2].

HAPE disrupts the balance between the production and return of intrapulmonary tissue fluid, causing a substantial amount of tissue fluid to extravasate from lung capillaries in a short period and accumulate in alveoli, lung interstitium, and bronchioles, thereby severely impairing lung ventilation and gas exchange functions [3]. Clinical symptoms include exertional dyspnea, cyanosis, paroxysmal cough with pink frothy sputum, and bilateral lung congestion with symmetrical wet rales [4]. Hypoxic pulmonary hypertension, a hallmark feature of HAPE, involves mechanisms such as excessive sympathetic activation, impaired nitric oxide (NO) synthesis, and excessive endothelin-1 (ET-1) synthesis. Increased pulmonary vascular pressure leads to damage to alveolar epithelial and capillary endothelial cells, resulting in protein-rich bronchoalveolar lavage fluid (BALF) containing red blood cells and inflammatory cells. Proteins that facilitate angiogenesis significantly impact vascular permeability. Animal studies have shown that vascular endothelial growth factor (VEGF) levels significantly increase under hypoxic conditions, leading to increased pulmonary vascular permeability and inducing HAPE [5,6]. Recent research indicates that sodium activation and water resorption in alveolar epithelial cells play a crucial role in the clearance of interstitial fluid in the alveoli, and hypoxia-induced increases in capillary pressure and endothelial-epithelial permeability allow fluid to enter the alveoli, triggering pulmonary edema [4].

Animal studies indicate that antioxidants such as polysaccharide extracts, puerarin, curcumin, silymarin, ginsenosides, or rhodiola extracts can prevent hypoxic pulmonary hypertension by regulating angiotensin II, protein kinase C pathways, inflammatory cell proliferation, an NF-κB signaling pathways [7,8,9,10,11]. However, the pathogenesis and molecular mechanisms of HAPE remain unclear. Currently, the primary treatment for HAPE patients involves inhaling high-concentration oxygen or low-concentration NO to reduce capillary permeability and promote the absorption of intrapulmonary extravasate; medications such as aminophylline, nifedipine, and dexamethasone are used to relieve bronchospasm, relax bronchial smooth muscles, strengthen the heart, and promote diuresis, but they are accompanied by strong toxic and side effects, including arrhythmia and coma [4]. The treatment options for HAPE are relatively limited and have limited efficacy. Failure to provide effective treatment can lead to secondary lung infections, shock, or death. Therefore, revealing and elucidating the pathogenic mechanisms and molecular mechanisms of HAPE, identifying key molecules involved in hypoxic stress, will effectively improve the diagnosis and treatment of HAPE, provide possible pathological bases and drug targets, and hold significant importance.

Aldose reductase (AKR1B1, AR), an important member of the AKR protein family, plays critical roles in nuclear receptor signal transduction, cell metabolism, inflammatory responses, osmoregulation, and other life activities [12]. As the first and rate-limiting enzyme in the polyol metabolic pathway, AR catalyzes the reduction of glucose to sorbitol, which is then converted to fructose by sorbitol dehydrogenase (SDH). Under normal physiological conditions, glucose is converted to glucose-6-phosphate by hexokinase and provides energy for the body through glycolysis and the tricarboxylic acid cycle, which is the main pathway of glucose metabolism. Recent studies have shown that some physiological or pathological factors can significantly upregulate AR expression and activity in tissues where they are normally low. When glucose concentrations are high, a large amount of glucose is metabolized through the polyol pathway, leading to the accumulation of sorbitol and fructose, the massive consumption of NADPH and NAD^+^, alterations in cellular osmolarity, and disruptions in cellular redox homeostasis [13]. Most research on AR has focused on diabetes and its complications due to its key position in the glucose metabolism pathway. However, the role of AR in HAPE has not been clearly reported.

Studies have indicated that inhibiting AR can downregulate ET-1 expression and alleviate endotoxin-induced pulmonary hypertension [14,15]; AR plays a crucial role in regulating pneumonic diseases and oxidative-stress-induced inflammatory damage [16,17]; inhibiting AR reduces the expression of HIF-1α and VEGF in hypoxia-induced colorectal cancer cells [18]; and AR participates in the regulation of the osmotic-stress-responsive transcription factor OREBP in response to osmotic stress [19]. These findings suggest that AR plays a significant role in oxidative stress and various diseases. Despite the extensive research on AR, the mechanisms underlying its regulation of oxidative stress homeostasis remain elusive. In this study, we aim to identify and investigate the mechanisms by which AR regulates hypoxic stress in lung epithelial cells, thereby enhancing our understanding of the pathogenesis of HAPE. Our findings could provide a possible pathological basis and novel drug targets for preventing and improving the diagnostic and therapeutic challenges associated with HAPE, which holds significant importance in clinical practice.

## 2. Results

### 2.1. Knockout of AR Alleviates Hypoxia-Induced Pulmonary Edema in Mice

**Figure 1:**
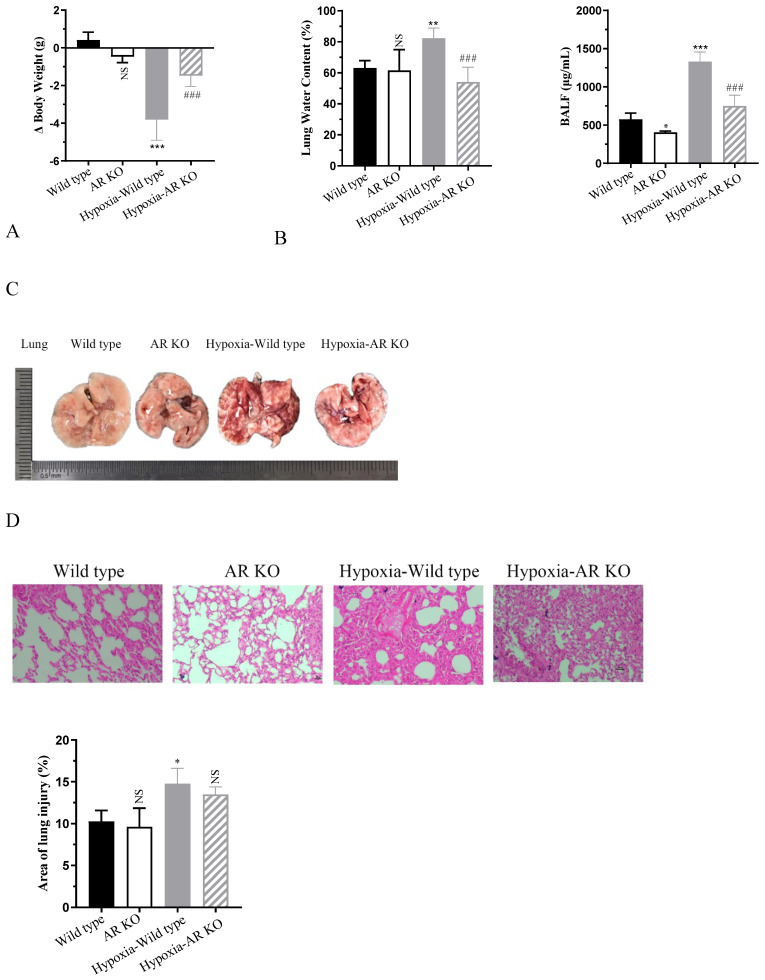

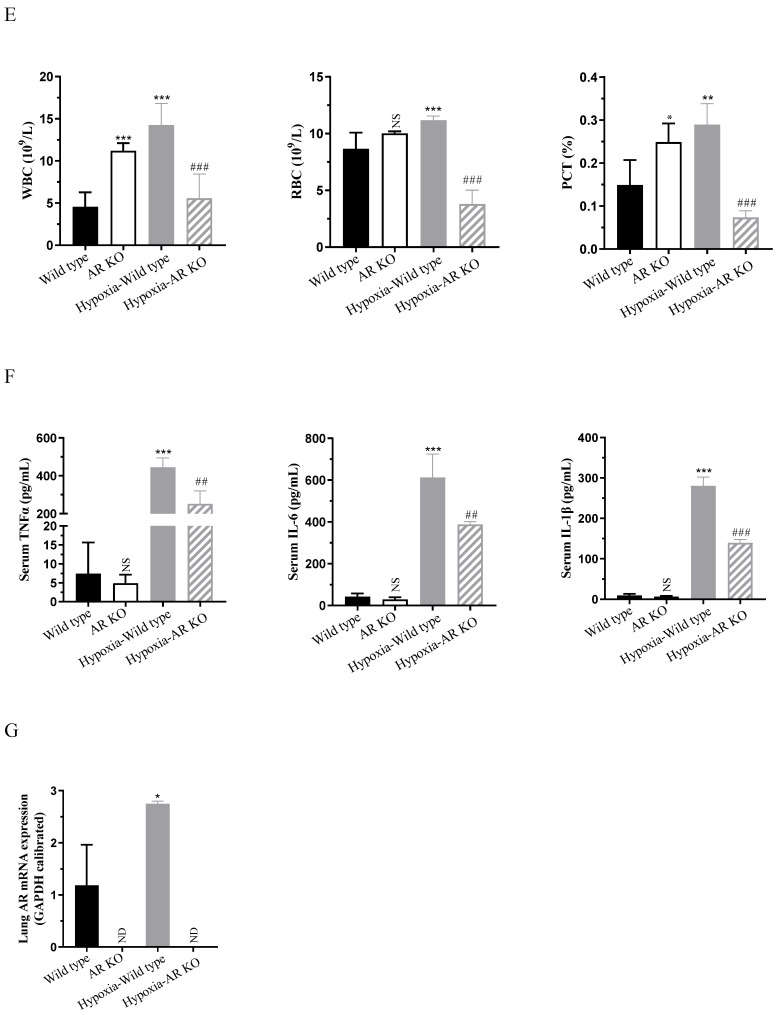
Mice were exposed to a low-pressure oxygen chamber simulating an altitude of 6000 m for 72 h. (**A**) Δ Body weight of mice. (**B**) Lung water content and BALF expression in mice. (**C**) Morphological analysis. (**D**) Hematoxylin and eosin (H&E) staining and quantitative statistics of lung injury. Scale bar: 100 px. (**E**) Blood routine of mice. (**F**) The secretion level of tumor necrosis factor-α (TNF-α), interleukin-6 (IL-6), and interleukin-1β (IL-1β). (**G**) The transcription level of AR. * *p* < 0.05, ** *p* < 0.01, and *** *p* < 0.001 vs. wild-type group; ^##^ *p* < 0.01 and ^###^ *p* < 0.001 vs. hypoxia-wild-type group. NS, not significant. ND, not detected.

To investigate the impact of aldose reductase (AR) in hypoxia-induced mice pulmonary edema, wild-type and *AR* KO mice were exposed to a low-pressure oxygen chamber simulating an altitude of 6000 m for 72 h. Notably, mice in the hypoxia group exhibited a significant decline in activity, often curling up and displaying accelerated breathing and decreased alertness. Compared to the normoxia group, the wild-type mice in the hypoxia group exhibited a significantly increased reduction in body weight, losing approximately 3.8 g (*p* < 0.0001). In contrast, the weight loss in *AR* KO mice was significantly attenuated, with a reduction of only about 1.4 g (*p* = 0.0004) (Figure 1A). Lung water content and BALF levels serve as indicators of the severity of lung edema. As shown in Figure 1B, after hypoxia, the lung water content and BALF levels of mice increased by approximately 1.3-fold (*p* = 0.0033) and 2.3-fold (*p* < 0.0001), respectively. However, *AR* KO mice exhibited a reduction in these hypoxia-induced increases by approximately 34.1% (*p* = 0.0005) and 43.6% (*p* < 0.0001). The morphological analysis of lung tissue revealed that lungs from normoxic mice were of normal size, with a dry, pale red surface devoid of congestion. Conversely, wild-type mice in the hypoxia group exhibited pronounced lung swelling and sclerosis, accompanied by bilateral lung bleeding and congestion, as well as red fluid exudation in the trachea. However, the *AR* KO mice in the hypoxia group had less bleeding on the surface of the lungs, and the degree of lung tissue damage was significantly reduced compared to the wild-type mice in the hypoxia group. H&E staining further demonstrated that the alveolar structure of normoxic mice remained clear and intact, with no edema or inflammatory cell infiltration in the alveoli or interstitium. Under hypoxic conditions, alveolar septa thickened significantly, and extensive bleeding and exudation occurred in the lung interstitium, accompanied by inflammatory cell infiltration, leading to near-complete disappearance of alveolar space. Notably, *AR* knockout significantly alleviated these phenomena (Figure 1C,D). The statistical analysis of the lung injury area revealed that, in comparison to the control group, hypoxia resulted in a notable 1.44-fold (*p* = 0.0301) increase in the area of lung injury. Conversely, the knockout of *AR* exhibited a beneficial effect by ameliorating the lung injury induced by hypoxia. The blood routine test results in Figure 1E demonstrated that, compared to the normoxic group, the levels of WBC, RBC, and PCT in the hypoxia group of mice increased significantly by approximately 3.1-fold (*p* < 0.0001), 1.3-fold (*p* < 0.0001), and 1.93-fold (*p* = 0.0037), respectively. However, *AR* KO reduced their levels by approximately 60.9% (*p* < 0.0001), 65.8% (*p* < 0.0001), and 74.6% (*p* = 0.0002), respectively. Similarly, Figure 1F indicated that the expression of inflammatory cytokines TNFα, IL-6, and IL-1β was significantly induced in the hypoxia group, with increases of approximately 60.1-fold (*p* < 0.0001), 14.3-fold (*p* < 0.0001), and 29.2-fold (*p* < 0.0001), respectively. *AR* KO inhibited their expression levels by approximately 43.3% (*p* = 0.0017), 36.7% (*p* = 0.0039), and 50.3% (*p* < 0.0001). Additionally, we examined the expression level of *AR*. Compared to the normoxia group, hypoxia significantly upregulated the mRNA expression of *AR* by approximately 2.5-fold (*p* = 0.0206), suggesting that AR may be involved in hypoxia-induced lung edema (Figure 1G).

### 2.2. AR Knockdown Ameliorates Hypoxia-Induced Expression of Vascular Pressure and Inflammatory Factors In Vitro

**Figure 2:**
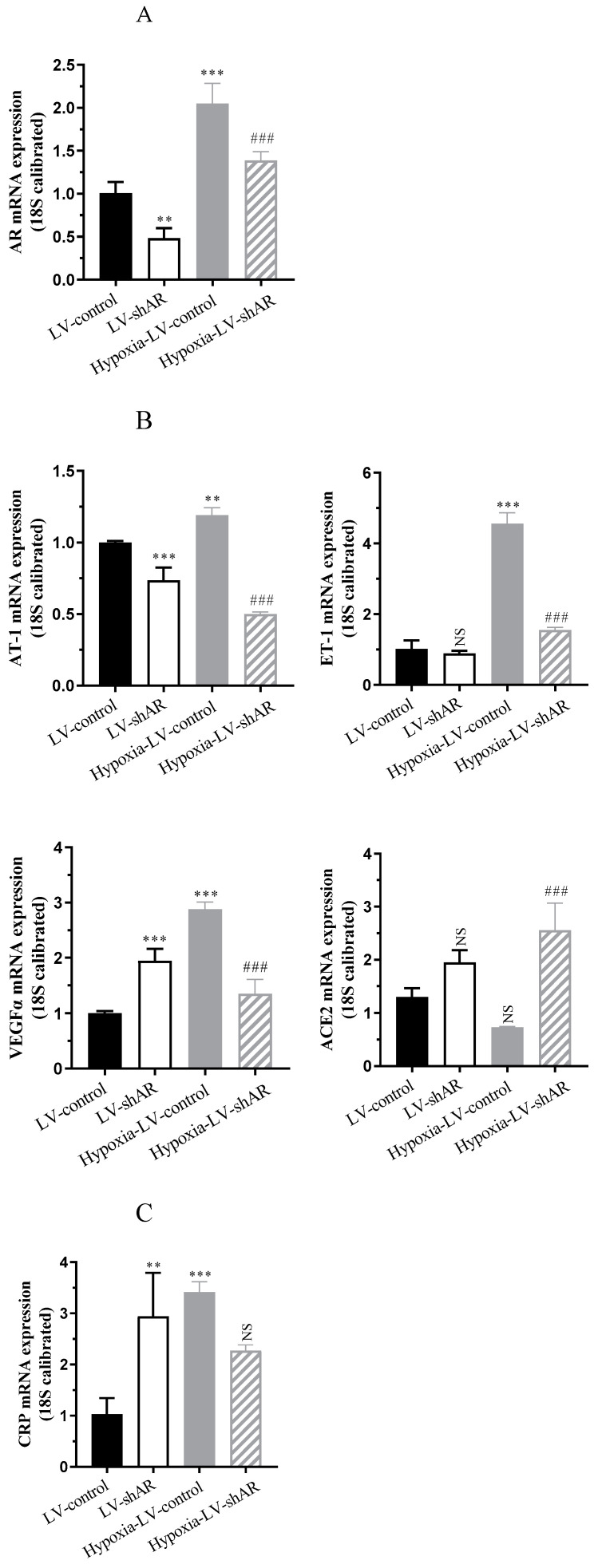

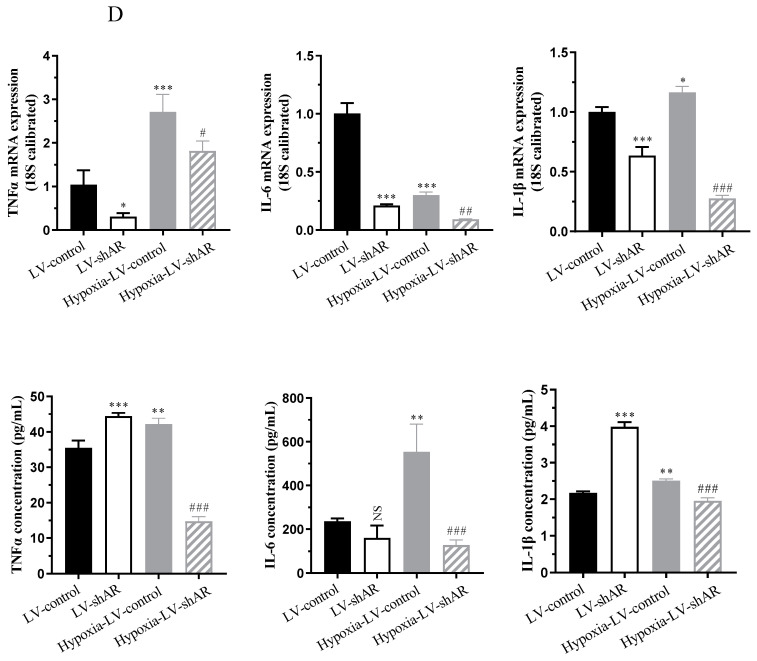
BEAS-2B cells with AR knockdown, along with control group cells, were subjected to hypoxia treatment. (**A**) The mRNA expression of *AR*. (**B**) The expression levels of angiotensin-1 (*AT-1*), endothelin-1 (*ET-1*), vascular endothelial growth factor (*VEGFα*), and angiotensin converting enzyme 2 (*ACE2*). (**C**) The mRNA expression of C-reactive protein (*CRP*). (**D**) The transcription and expression levesl of TNF-α, IL-6, and IL-1β. * *p* < 0.05, ** *p* < 0.01, and *** *p* < 0.001 vs. LV-control group; ^#^ *p* < 0.05, ^##^ *p* < 0.01, and ^###^ *p* < 0.001 vs. hypoxia-LV-control group. NS, not significant.

We initially evaluated the efficacy of *AR* knockdown in BEAS-2B cells post lentiviral infection, as illustrated in Figure 2A, compared to the control group, the mRNA expression of *AR* was reduced by approximately 50% (*p* = 0.0013). According to our previous research, 800 μM Cocl_2_ was used to treat BEAS-2B cells for 24 h to construct an in vitro hypoxia model [20]. Additionally, hypoxia was found to stimulate an approximate 2-fold (*p* < 0.0001) increase in *AR* expression at the cellular level. Subsequently, we examined the expression of factors related to vascular pressure. Notably, under hypoxia conditions, the mRNA expression of *AT-1* (increased by approximately 1.2-fold, *p* = 0.0067), *ET-1* (increased by approximately 4.6-fold, *p* < 0.0001), and *VEGFα* (increased by approximately 2.9-fold, *p* < 0.0001) were significantly upregulated, while the activity of angiotensin converting enzyme 2 (*ACE2*) decreased by approximately 46.2% (*p* = 0.1203), leading to an increase in vascular pressure. However, when *AR* was knocked down, all of the aforementioned factors exhibited significant alleviation (Figure 2B).

Under hypoxia conditions, the hypoxia-inducible factor within cells becomes activated and plays a pivotal role in regulating essential processes including cellular glucose metabolism, angiogenesis, and erythropoiesis. Furthermore, it modulates the expression of downstream genes that are intricately linked to inflammation and immune responses. As depicted in Figure 2C,D, hypoxia stimulation led to an approximately 3.4-fold (*p* = 0.0007) increase in C-reactive protein (*CRP)* transcription, a 2.7-fold (*p* = 0.0003) increase in *TNF-α*, and a 1.2-fold (*p* = 0.0116) increase in *IL-1β*. However, these increases were inhibited when *AR* was knocked down.

### 2.3. Knocking Down AR Improves the Expression of Oxidative Stress Induced by Hypoxia

**Figure 3:**
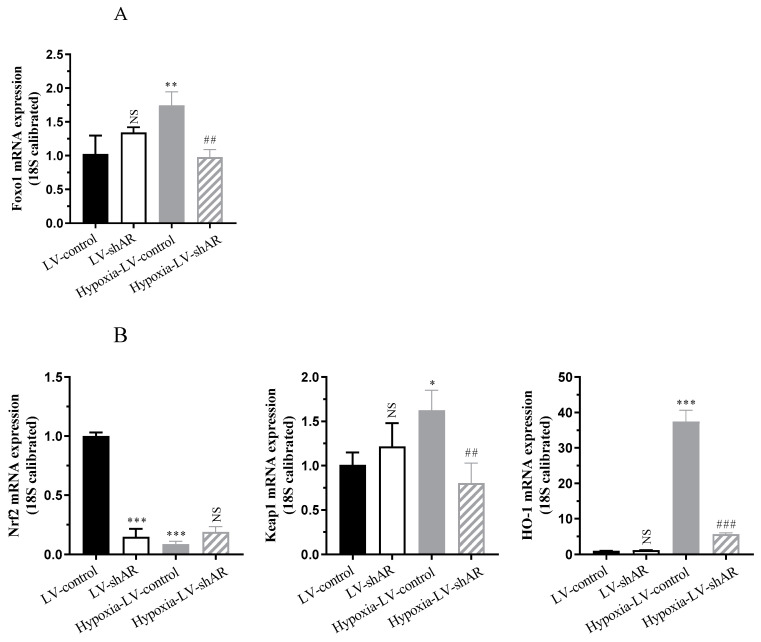

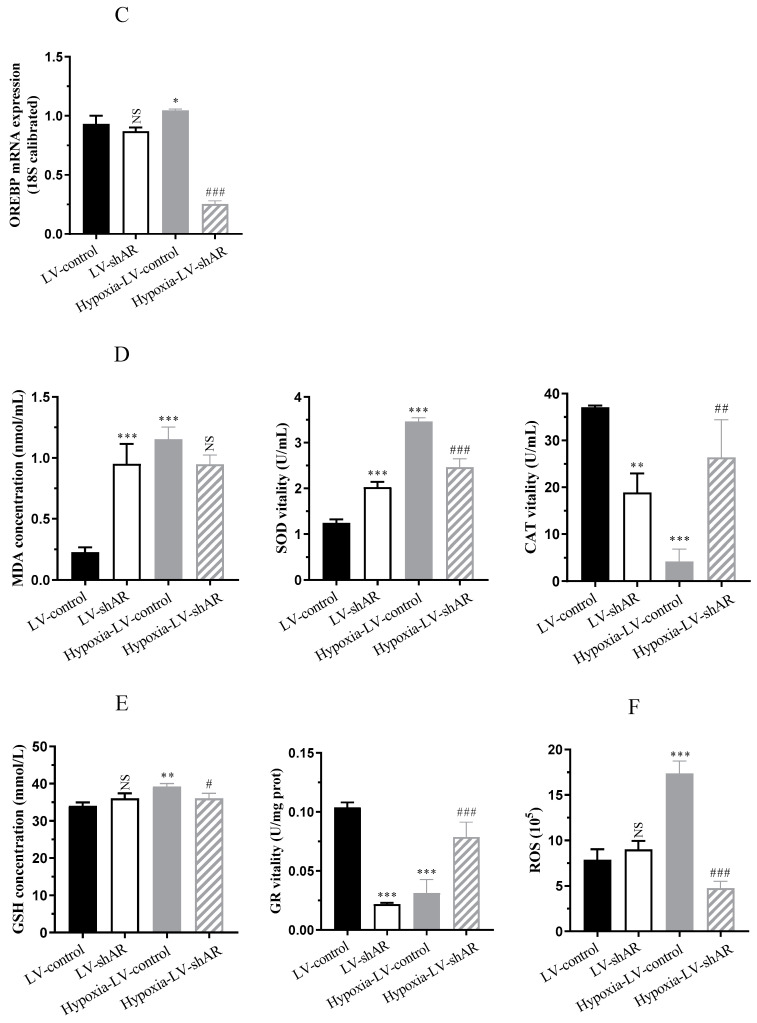
(**A**) The mRNA expression of Forkhead box protein o1 (*Foxo1*). (**B**) The mRNA expression of nuclear factor erythroid 2-related factor 2 (*Nrf2*), kelch-like ECH-associated protein 1 (*Keap1*), and heme oxygenase 1 (*HO-1*). (**C**) The mRNA expression of osmotic response element-binding protein (*OREBP*). (**D**) The activity of malondialdehyde (MDA), superoxide dismutase (SOD), and catalase (CAT). (**E**) The activity of glutathione (GSH) and glutathione reductase (GR). (**F**) The content of reactive oxygen species (ROS). * *p* < 0.05, ** *p* < 0.01, and *** *p* < 0.001 vs. LV-control group; ^#^ *p* < 0.05, ^##^ *p* < 0.01 and ^###^ *p* < 0.001 vs. hypoxia-LV-control group. NS, not significant.

Forkhead box protein o1 (Foxo1), a key regulator of oxidative stress, was upregulated after hypoxia [21]. Conversely, nuclear factor erythroid 2-related factor 2 (Nrf2) expression was downregulated by hypoxia, while kelch-like ECH-associated protein 1 (Keap1), which negatively binds to Nrf2, showed an upregulation trend due to decoupling from Nrf2 under oxidative stress conditions [21,22]. Heme oxygenase 1 (HO-1), as a downstream gene of Nrf2, exhibited antioxidant and anti-inflammatory effects [23]. Additionally, the osmotic response element-binding protein (OREBP) was activated in hyperosmotic environments [24]. The current study investigated the involvement of AR in the redox system and antioxidant regulation under hypoxic stress. The knockdown of *AR* mitigated the expression of oxidative stress factors, as evidenced by a reduction in *Foxo1* by approximately 43.9% (*p* = 0.0027), an upregulation of *Nrf2* by roughly 2.1-fold (*p* = 0.0612), a decrease in *Keap1* by about 50.6% (*p* = 0.0052), a significant drop in *HO-1* by approximately 84.9% (*p* < 0.0001), and a decrease in *OREBP* by around 75.8% (*p* < 0.0001). These findings affirm the involvement of AR in the redox system and antioxidant regulation under hypoxic stress conditions, as illustrated in Figure 3A–C.

Studies have shown that superoxide dismutase (SOD) and catalase (CAT) are the main enzymes responsible for scavenging free radicals in the body and are important indicators of hypoxia resistance [25,26]. Figure 3D revealed that following 6 h of cellular hypoxia, there was a marked upregulation of malondialdehyde (MDA) by roughly 5.0-fold (*p* < 0.0001) and SOD by approximately 2.8-fold (*p* < 0.0001). Conversely, CAT activity and antioxidant capacity decreased by nearly 88.6% (*p* < 0.0001). Notably, the oxidative stress was mitigated upon knocking out AR. The depletion of glutathione (GSH) in tissues occurs under conditions such as hypoxia and inflammatory stimuli. With the depletion of intracellular GSH, glutathione reductase (GR) reaction cannot metabolize lipid peroxides, resulting in weakened cellular antioxidant capacity and disruption of intracellular redox balance [27,28]. As depicted in Figure 3E, after 6 h of exposure to hypoxia, GSH level increased by 1.2-fold (*p* = 0.0053), whereas GR activity decreased by approximately 70% (*p* < 0.0001), indicating a response to oxidative stress. Upon knocking out AR, the cells were able to recover from the oxidative stress state. Additionally, Figure 3F shows that, in comparison to the normoxic control group, the cellular content of reactive oxygen species (ROS) significantly increased by approximately 2.2-fold (*p* < 0.0001) after 6 h of hypoxia. The subsequent decrease in ROS levels upon knocking out AR further substantiates the role of AR in oxidative stress responses during hypoxic conditions.

### 2.4. Verify the Regulatory Role of AR on Various Physiological Indicators Under Hypoxic Stimulation at the Protein Expression Level

**Figure 4:**
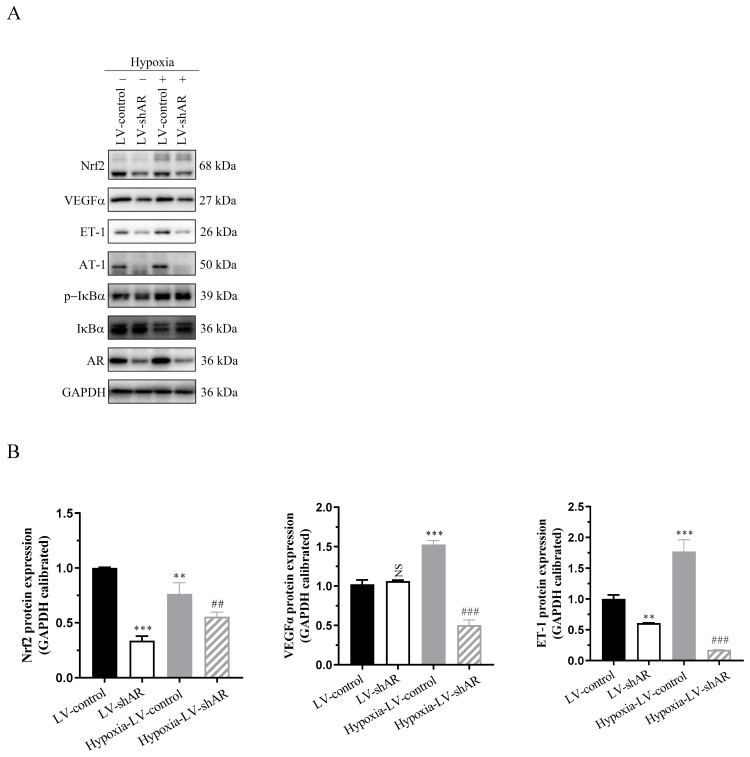

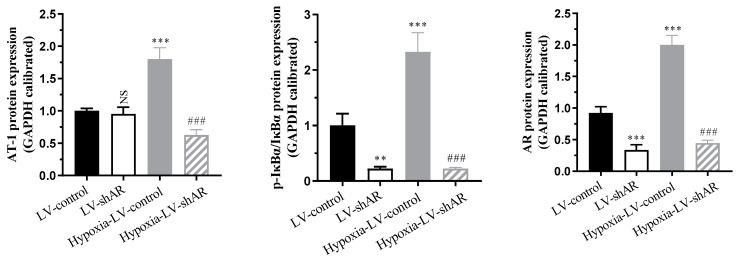
(**A**) The protein expression of related proteins. (**B**) Statistical analysis results of (**A**). ** *p* < 0.01 and *** *p* < 0.001 vs. LV-control group; ^##^ *p* < 0.01 and ^###^ *p* < 0.001 vs. hypoxia-LV-control group. NS, not significant.

As shown in Figure 4, the protein expression of AR is significantly induced by hypoxia. After knocking down AR, the expression trends of various proteins involved in cellular hypoxic stress, including VEGFα, ET-1, AT-1, p-IκBα/IκBα, and Nrf2, are consistent with our previously reported results. This further verifies that AR participates in the regulation of high-altitude pulmonary edema (HAPE) pathogenesis by modulating vascular pressure factors, inflammatory factors, oxidative stress factors, and other factors under hypoxic stimulation.

### 2.5. AR Regulation of Blood Glucose and Lactate Accumulation Under Hypoxic Conditions

**Figure 5:**
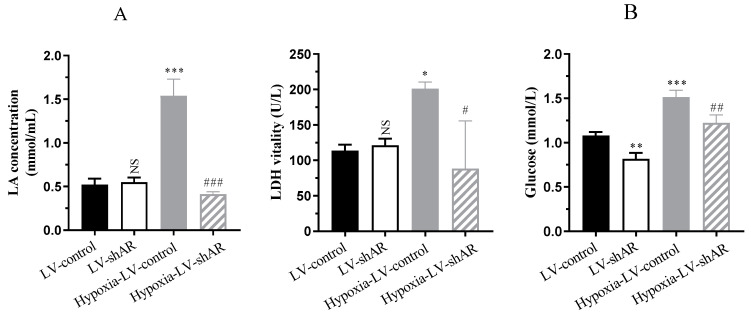
(**A**) The content of lactic acid (LA) and the activity of lactate dehydrogenase (LDH). (**B**) The level of glucose. * *p* < 0.05, ** *p* < 0.01, and *** *p* < 0.001 vs. LV-control group; ^#^ *p* < 0.05, ^##^ *p* < 0.01, and ^###^ *p* < 0.001 vs. hypoxia-LV-control group. NS, not significant.

Studies have demonstrated that at high altitudes, hypoxic conditions accelerate metabolism and elevate blood glucose concentrations [29,30]. Consequently, we investigated the impact of AR on blood glucose and lactate accumulation under hypoxic conditions. Our findings demonstrated that hypoxic stimulation markedly elevated lactate accumulation from 0.5 mmol/mL to 1.5 mmol/mL (*p* < 0.0001), accompanied by a 1.8-fold (*p* = 0.0438) augmentation in lactate dehydrogenase (LDH) activity, and glucose levels augmented from 1.1 mmol/L to 1.5 mmol/L (*p* = 0.0002). Remarkably, subsequent to knocking out *AR*, the concentrations of these molecules decreased in comparison to their levels prior to the knockout (Figure 5). These results affirm that AR regulates blood glucose and lactate accumulation in response to hypoxic stimulation, thereby accelerating metabolic processes in the body. Our research indicates that AR plays a pivotal role in metabolic adaptation to hypoxia conditions and holds potential as a therapeutic target for managing metabolic disorders linked to hypoxia.

## 3. Discussion

High-altitude pulmonary edema (HAPE) poses a significant health risk due to its sudden onset, rapid progression, and high mortality rate among those rapidly exposed to high altitudes. The incidence of HAPE is positively correlated with altitude and the rate of ascent; higher altitudes and faster ascent rates significantly increase the risk, with an incidence estimated to range from 0.01% to 15.5% [31,32]. As altitude increases, atmospheric pressure decreases, particularly the oxygen partial pressure inhaled by humans, significantly impairing the transfer of oxygen from pulmonary veins to pulmonary arteries [33]. HAPE manifests with symptoms such as decreased physical endurance, exertional dyspnea, tachypnea, and cough. If not treated promptly, the condition can worsen, leading to orthopnea and, in some cases, fever, with a mortality rate as high as 50% [1]. Current research on HAPE’s pathogenesis primarily focuses on five aspects: hypoxic pulmonary artery hypertension, altered vascular permeability, reduced alveolar liquid clearance, oxidative stress, and inflammatory responses [34,35]. Therefore, exploring new therapeutic targets is of great significance for the treatment of HAPE.

The results presented in this study suggest that aldose reductase (AR) may be a crucial target in addressing hypoxia-induced pulmonary edema and related metabolic and inflammatory disturbances. By exposing both wild-type and *AR* KO mice to low-pressure oxygen conditions simulating an altitude of 6000 m, we observed a significant alleviation of pulmonary edema severity in *AR* KO mice compared to wild-type mice. This was evident from the reduced weight loss, lung water content, and BALF levels in *AR* KO mice. Morphological analysis further confirmed these findings, showing less pronounced lung swelling, sclerosis, and bleeding in *AR* KO mice exposed to hypoxia.

HAPE is a non-cardiogenic pulmonary edema, with high-altitude hypoxic pressure being the primary inducer. Upon arrival at high altitudes, the amount of alveolar fluid depends on the liquid extravasation from pulmonary blood vessels, primarily related to hypoxic pulmonary hypertension, and the rate of alveolar epithelial fluid reabsorption, mainly determined by sodium transport in alveolar epithelia [36]. Hypoxic pulmonary hypertension, a hallmark of HAPE, involves mechanisms such as the overactivation of the sympathetic nervous system, excessive ET-1 synthesis. Elevated pulmonary vascular pressure can damage alveolar epithelial and capillary endothelial cells, ultimately leading to the accumulation of protein-rich, erythrocyte-laden, and inflammatory cell-containing BALF. Consequently, certain proteins that contribute to angiogenesis have a significant impact on vascular permeability. Animal studies have shown that VEGF significantly increases under hypoxic conditions, enhancing lung vascular permeability and inducing HAPE [37]. Additional studies suggest significant increases in IL-6 and CRP in HAPE patients, indicating the involvement of inflammation in the pathogenesis of HAPE [38].

Moreover, our in vitro studies using BEAS-2B cells with *AR* knockdown corroborated the in vivo findings. Hypoxia-induced increases in *AR* expression were accompanied by alterations in vascular pressure-related factors, such as *AT-1*, *ET-1*, *VEGFα*, and *ACE2*. These changes, leading to increased vascular pressure, were alleviated upon *AR* knockdown. Additionally, the modulation of inflammatory factors, including *TNF-α*, *IL-6*, and *IL-1β*, and the increase in *CRP* transcription were also inhibited when *AR* was knocked down. These results confirm the regulatory role of AR in both vascular pressure and inflammatory responses under hypoxic conditions. These findings suggest that AR plays a pivotal role in regulating these factors under hypoxic conditions.

AR, a key enzyme in the polyol pathway, targeting AR may ameliorate pulmonary artery hypertension, reduce the expression of hypoxia-inducible factors and vascular endothelial growth factors, alleviate inflammatory responses, and improve osmotic stress. Several mainstream scientific hypotheses have been proposed to explain the involvement of AR in oxidative stress: (1) Osmotic stress: AR-catalyzed overaccumulation of sorbitol can lead to electrolyte imbalance, causing cellular water influx and swelling, ultimately resulting in cellular damage [39]. (2) Oxidative stress: the depletion of NADPH competitively inhibits NOS and GR, both of which require NADPH as a cofactor. With NADPH depletion, GR activity is inhibited, resulting in reduced levels of reduced GSH, thereby decreasing the cellular antioxidant capacity and impairing the redox system [40]. (3) Pseudo-hypoxia: increased AR activity elevates the NADH/NAD^+^ ratio, leading to a state of “pseudo-hypoxia”, which mimics the conditions of hypoxia and increases oxidative stress responses, causing cellular and tissue damage. (4) Protein kinase C (PKC) activation: by regulating the activity of related enzymes and cytokines, PKC influences physiological metabolic activities [41]. Our study demonstrated that *AR* knockdown improved the antioxidant capacity of cells exposed to hypoxia. This was evident from the knockdown of *AR* mitigated the expression of oxidative stress factors, such as *Foxo1* and *Keap1*, while upregulating *Nrf2*. Concurrently, the antioxidant enzymes SOD and CAT, as well as GSH and GR activities, were modulated in response to hypoxia. Notably, the depletion of *AR* improved the cells’ ability to recover from oxidative stress, as evidenced by decreased ROS levels. These findings affirm the involvement of AR in the redox system and antioxidant regulation under hypoxic stress conditions.

Meanwhile, our study revealed that AR regulates blood glucose and lactate accumulation in response to hypoxic stimulation. Hypoxia-induced elevations in lactate, LDH activity, and glucose levels were decreased upon *AR* knockout. In conclusion, our results suggest that AR may play a pathogenic role in hypoxia-induced pulmonary edema by regulating vascular pressure, inflammation, oxidative stress, and metabolic processes. AR may be a potential therapeutic target for the prevention and treatment of hypoxia-induced pulmonary edema.

## 4. Materials and Methods

### 4.1. Reagents

BEAS-2B cells were obtained from Central China for type culture collection. Cobalt chloride (Cocl_2_) was sourced from Kermel chemreagent Co., Ltd. (Tianjin, China). ELISA kits for human or mouse interleukin-6 (IL-6), human or mouse tumor necrosis factor-α (TNFα), and human or mouse interleukin-1β (IL-1β) were obtained from Boster biological technology., Ltd. (Wuhan, China). Primary antibodies against angiotensin-1 (AT-1) and endothelin-1 (ET-1) were purchased from Abclonal (Wuhan, China). Primary antibodies for GAPDH, IκBα, phospho-IκBα, AR, vascular endothelial growth factor (VEGFα), and Nrf2 were purchased from Sangon biotech (Shanghai, China). HRP-conjugated goat anti-rabbit IgG was purchased from Sangon biotech (Shanghai, China).

### 4.2. Animal Treatment

Male C57BL/6 wild-type mice and aldose reductase knockout (*AR* KO) mice, both aged 10 weeks and weighing between 20 and 25 g, which had been reared in our laboratory for an extended period and were originally sourced from the Jackson Laboratory in the United States, were maintained under a controlled 12/12 h light/dark cycle with unrestricted access to food and water. Subsequently, the mice were randomly allocated into four groups, with six mice in each group: (1) normoxic wild type, (2) normoxic *AR* KO, (3) hypoxia wild type, and (4) hypoxia *AR* KO. The hypoxic condition was as follows: exposure to a low-pressure oxygen chamber simulating an altitude of 6000 m for 72 h.

### 4.3. Lung Water Content

The water content within lung tissue serves as a critical metric for evaluating the extent of pulmonary edema. To assess this, the middle lobe of the right lung is carefully handled to remove surface liquids using filter paper, after which its wet weight (W) is promptly determined and documented. Concurrently, the lower lobe of the right lung undergoes drying in an oven set at 56 °C for a period of 72 h until achieving a stable dry weight (D). The lung water content (LWC) is subsequently calculated with the formula: LWC (%) = (W − D)/W × 100%. This percentage provides insight into the severity of lung edema present in mice.

### 4.4. Bronchoalveolar Lavage Fluid

To collect bronchoalveolar lavage fluid (BALF), the distal end of the trachea is ligated, and the lungs are gently lavaged 2–3 times with 1 mL of pre-cooled saline. The BALF is then carefully aspirated and subjected to centrifugation at 4 °C for 10 min at a speed of 3500 rpm. Following centrifugation, the supernatant is harvested for analysis. The total protein concentration within the BALF is quantified using a bicinchoninic acid (BCA) protein assay kit.

### 4.5. Hematoxylin and Eosin Staining

Lung tissues were immediately dissected and fixed in a 4% paraformaldehyde solution for 24 h, followed by paraffin embedding. Sections were prepared and stained following standard histological protocols. Hematoxylin and eosin (H&E) staining was performed on the sections to facilitate the examination of pathological changes under a Nikon light microscope.

### 4.6. Cell Culture

Human pulmonary bronchial epithelial cells (BEAS-2B) were maintained at 37 °C in a humidified atmosphere containing 5% CO_2_. The culture medium consisted of MEM supplemented with 10% fetal bovine serum and antibiotics (100 IU/mL streptomycin and 100 IU/mL penicillin). For gene knockdown experiments, HEK-293T cells were transfected with lentiviral vectors using a lentiviral system to produce AR-specific short hairpin RNA (shRNA) lentiviruses. These lentiviruses were then used to infect BEAS-2B cells, generating AR knockdown cells (lentiviral vector (LV)-shAR) and their respective controls (LV-control). Data were collected from three independent cell cultures.

### 4.7. Enzyme-Linked Immunosorbent Assay

To determine the concentration of inflammatory factors, mouse serum or cell supernatants were analyzed using enzyme-linked immunosorbent assay (ELISA) kits according to the manufacturer’s instructions. Absorbance was measured at 450 nm to quantify the results.

### 4.8. Real-Time PCR

Total RNA extraction from lung tissue or BEAS-2B cells was performed using TRIzol reagent (Invitrogen, Waltham, MA, USA), strictly following the manufacturer’s protocol. A total of 1 μg of total RNA was reverse-transcribed into cDNA using the ReverTrace kit (Takara Bio, Beijing, China). Real-time quantitative PCR was conducted using SYBR green as the fluorescent dye on a CFX96 Connect instrument (Bio-Rad, Hercules, CA, USA).

### 4.9. Western Blot

Proteins from lung tissue or cell lysates were separated by 10% SDS-polyacrylamide gel electrophoresis and transferred onto polyvinylidene difluoride (PVDF) membranes. Membranes were blocked with 5% bovine serum albumin (BSA) for one hour at room temperature before being incubated overnight at 4 °C with specific primary antibodies. After washing, membranes were incubated with corresponding secondary antibodies for an additional hour at room temperature. To ensure reproducibility, all Western blot experiments were repeated at least three times independently. Band density was quantified using ImageJ software (Image-Pro Plus Version 6.0) and normalized against the internal control GAPDH.

### 4.10. Statistical Analysis

Experimental data are presented as mean ± standard deviation. Normality was assessed using the Anderson–Darling tests. Post-hoc and multiple comparison tests were applied for further analysis. A one-way analysis of variance (ANOVA) was utilized to evaluate the statistical significance of differences among groups.

## 5. Limitations

While our study presents compelling evidence for the role of aldose reductase (AR) in hypoxia-induced pulmonary edema, it is not without limitations. Firstly, the sample size used in our animal and cell culture experiments was relatively small, which may limit the generalizability of our findings. Larger sample sizes would be required to confirm our observations and to explore potential sex-specific differences in AR-mediated responses to hypoxia. Secondly, our study focused on the acute effects of hypoxia on AR-mediated responses. It would be interesting to investigate the chronic effects of hypoxia on AR expression and function, as well as the potential involvement of other signaling pathways in regulating these responses. Additionally, while our findings suggest that AR is a potential therapeutic target for managing metabolic disorders linked to hypoxia, further studies are required to validate this hypothesis in clinical settings. It would also be important to explore the potential side effects of inhibiting AR activity in humans and to develop safe and effective inhibitors for therapeutic use.

## 6. Conclusions

In summary, our study demonstrates that aldose reductase (AR) plays a critical role in regulating the responses to hypoxia-induced high-altitude pulmonary edema (HAPE). By knocking out or knocking down AR, we observed a significant alleviation of pulmonary edema severity, reductions in vascular pressure and inflammatory factors, improvements in antioxidant capacity, and reductions in blood glucose and lactate accumulation. These findings could provide a potential pathological basis and novel drug targets for preventing and improving the diagnostic and therapeutic challenges associated with HAPE, which holds immense significance in clinical practice.

## Data Availability

The datasets in the current study are included in the published article or available from the corresponding author on reasonable request.
